# Competitive Exclusion of Flavescence dorée Phytoplasma Strains in *Catharanthus roseus* Plants

**DOI:** 10.3390/plants9111594

**Published:** 2020-11-17

**Authors:** Marika Rossi, Marta Vallino, Luciana Galetto, Cristina Marzachì

**Affiliations:** CNR—Institute for Sustainable Plant Protection, 10135 Torino, Italy; marika.rossi@ipsp.cnr.it (M.R.); marta.vallino@ipsp.cnr.it (M.V.); luciana.galetto@ipsp.cnr.it (L.G.)

**Keywords:** *Vitis vinifera*, 16SrV-C, 16SrV-D, ‘*Candidatus* Phytoplasma vitis’, competition

## Abstract

Flavescence dorée phytoplasmas (FDp, 16SrV-C and -D) are plant pathogenic non-cultivable bacteria associated with a severe grapevine disease. The incidence of the two reference strains on cultivated grapevines is unbalanced, and mixed infections are rare. To investigate the interaction between the two strains, *Catharanthus roseus* plants were graft-infected with both strains, either simultaneously or sequentially. Different combinations of lateral and apical grafting were applied to avoid possible benefits due to graft position. The infection was monitored for four months through a new diagnostic protocol developed for differentiation and relative quantification of the two strains. Regardless of the temporal or spatial advantage at grafting, FD-C generally outcompeted FD-D. The prevalence of FD-C increased over time and, at the end of the experiment, FD-C was the unique strain detected in the aerial part and the roots of 74% and 90% of grafted plants, respectively. These data indicate that the interaction between the two strains results in competitive exclusion. Understanding the bases of the competition between FD-C and FD-D may contribute to explain the biology of the coexistence of different FDp strains under field conditions, aiming at identifying potential suppressor strains, which can provide alternative and environmentally sustainable solutions for FD control.

## 1. Introduction

Flavescence dorée (FD) of grapevine, is a quarantine disease in Europe caused by the FD phytoplasma (FDp), a wall-less and uncultivable mollicute transmitted mainly by the monovoltine leafhopper *Scaphoideus titanus* Ball in a persistent and propagative way [1,2]. The vector was introduced from North America into Europe in the 1950s [3], while the European origin of the phytoplasma has been recently demonstrated [4]. Outbreaks of FD caused by the contemporaneous presence of the phytoplasma and its vector is posing environmental threats due to compulsory insecticide control of the insect, and hamper the sustainability of viticulture in the most important European grapevine growing districts [5]. The FDp belongs to the 16SrV-C and -D taxonomic subgroups [6,7,8,9], of the proposed ’*Candidatus* Phytoplasma vitis’ species [10], which has not been formally described yet. Both strains are transmitted by *S. titanus* [11], and share similar pathogenicity and symptomatology, as no differences in aggressiveness is reported either in grapevine or in the experimental periwinkle, broad bean, and *Arabidopsis thaliana* hosts. Genetic characterization of FDp populations in a wine-growing region in Italy revealed that the -D subgroup was present in more than 70% of the disease cases, and the incidence of the -C strain was higher in plants than in insects [12]. Moreover, despite the presence in the same vineyard of both FDp strains, mixed FD-C + FD-D infections were less than 5% of the total disease cases [12]. In nature, plants can be infected by different pathogens simultaneously, and mixed infection by closely or distantly related phytoplasmas are quite common [13]. Surprisingly, mixed infections with the two FDp strains are rare in the vineyard [14,15,16]. Differences in the incidence of two pathogen strains in a host and low mixed infection rates could be due to competitive exclusion, a form of competition between species (or strains) that leads to the elimination of one of the competitors from a given habitat [17]. Aim of this work was to confirm the existence of FD-C vs. FD-D strain competition in the plant. In particular, the possible competition was studied upon controlled graft inoculation of periwinkle, a laboratory host of the phytoplasma. The presence and the load of the FD-C and -D phytoplasma strains were monitored, with a newly developed quantitative Real Time PCR protocol, at several time points in doubly inoculated plants with different grafting combinations. Indeed, the multiplication kinetics of FD-C and FD-D strains in single infection is similar in the broad bean laboratory host, and the phytoplasma load at the end of the infection is comparable [18]. Analogously, FD-C and FD-D loads in grapevine plants of two genotypes with single infection of the two strains are similar [19].

Management of FD epidemics relies on the understanding of the epidemiological cycle of the disease, involving the ampelophagous *S. titanus* as well as other occasional phloem feeder vectors [4,20,21], and wild alternative host plants for the phytoplasma [16,22]. The existence of competing FDp strains would add more complexity to the epidemiology of this disease, and help fine tuning of control strategies.

## 2. Results

### 2.1. Development of a Real Time PCR Protocol for Simultaneous Diagnosis and Quantification of FD-C and -D Strains

Three genomic portions of FDp were selected to optimize a method for rapid and simultaneous detection and quantification of the two strains: the ribonucleoside-diphosphate reductase 2 β subunit (*nrd*F), the maltose ABC transporter membrane subunit (*mal*G), and the group II intron gene (contig 12). These sequences were screened as targets for the development of the new diagnostic protocol based on the discrimination of the melting peak of the amplicons from each FDp isolate. The *mal*G gene showed about 99% sequence identity between FD-C and the most frequent FD-D genotypes, including the malG1 of the FD-D isolate used in this work. The *nrd*F gene showed 98% sequence identity between -C and -D phytoplasmas, corresponding to 21 SNPs. The contig 12 showed 98.6% sequence identity between FD-C and -D strains (8 SNPs and 2 insertions/deletions). Primer pairs were designed on each of the three genes to include most of the SNPs between the two strains, as detailed in Table 1. The analysis of the melting peaks produced by the amplification of the three gene portions from the FD-C and -D phytoplasmas showed that the melting temperatures were too close to allow reliable discrimination of the two strains, when present in dual infections in the same plant (Table 1). Therefore, a duplex Real Time PCR on *nrd*F gene was developed. A single primer pair (NrdF_F28/NrdF_R121), matching both FD-C and -D sequences, and two strain-specific TaqMan probes were designed on a portion displaying two SNPs between the two strains (Table 1). Upon single infection with each FDp strain, both probes were highly specific for their targets and showed no cross-recognition or non-specific signal from negative controls (data not shown). Both standard curves showed acceptable R^2^ values and reaction efficiencies (Table 1). The detection limit for both -C and -D templates was 10 genome units (GU) of FDp. Moreover, the protocol was able to detect both strains artificially mixed up to 7.5 ratio, confirming the reliability of this protocol to discriminate between the two FDp strains when present simultaneously in the same plant.

### 2.2. FD-C and FD-D Infections in Catharanthus roseus

**Figure 1:**
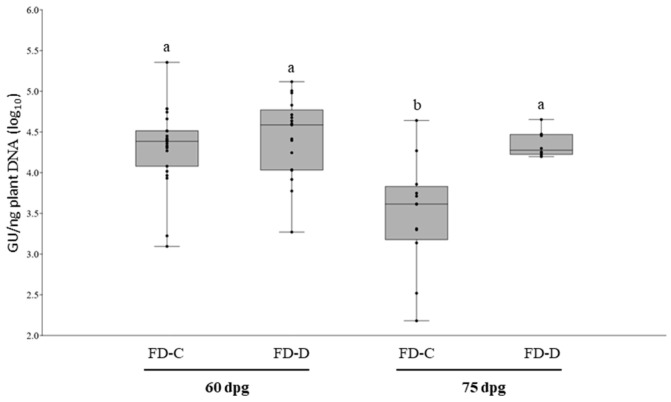
Boxplot with jitter of Flavescence dorée (FD) phytoplasma loads in periwinkle leaves of infected plants at 60 and 75 days post grafting (dpg) with FD-C or -D infected scions. Each dot represents one sample. Different letters indicate significant differences in phytoplasma load (ANOVA; F(3, 56) = 9.687; *p* = 3.015 × 10^−5^).

The pathogenicity and the infection dynamic of each of the two FDp strains in shoots and roots were studied with two separate experimental inoculations of *C. roseus* plants. Two sets of 26 *C. roseus* plants were grafted with either FD-C or FD-D infected scions. At symptoms appearance (60 days post grafting, dpg) 88.5% and 65.4% of the inoculated plants showed symptoms and tested PCR positive for FD-C and for FD-D respectively. The remaining FD-C and FD-D grafted plants were asymptomatic and PCR negative. The average phytoplasma load was similar between the two treatments and ranged between 1.24 × 10^3^ and 2.27 × 10^5^ GU/ng of total plant DNA (Figure 1). At 75 dpg, FD-Cp average load decreased to 7.87 × 10^3^ (standard error of the mean (SEM) ± 5.56 × 10^3^), and it was significantly lower than that of FD-Dp (2.38 × 10^4^ ± 3.57 × 10^4^) (*p* < 0.05) (Figure 1). No differences were observed in the type of symptoms or their severity throughout the experiment (data not shown).

**Figure 2:**
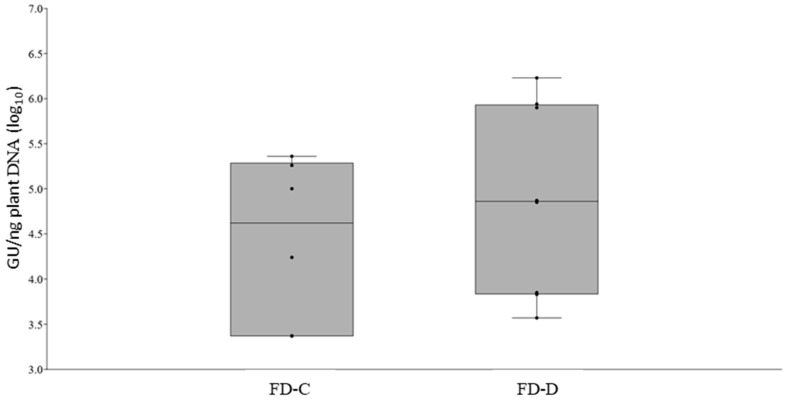
Boxplot with jitter of Flavescence dorée (FD) phytoplasma loads in periwinkle roots of infected plants at 12 days post grafting with FD-C or -D infected scions (*t*-test, *p* = 0.212). Each dot represents one sample.

To study the dynamic of root colonization of each phytoplasma, two sets of 15 periwinkle plants were grafted with either FD-C or FD-D infected scions. Every four days starting from the grafting, roots were collected from three plants of each treatment to determine the earliest date to detect phytoplasma presence in that tissue. FDp was clearly detectable by qPCR at 12 dpg in 5 out of 6 tested plants and therefore all the remaining plants were sampled at that date. Overall, at that date, FD-C and -D phytoplasmas were detected in the root systems of six and eight plants (out of the nine analyzed for each strain), and the measured phytoplasmas loads were similar (*t*-test; *p* = 0.212) ranging between 2.32 *×* 10^3^ and 1.69 *×* 10^6^ GU/ng plant DNA (Figure 2, Table S1).

**Figure 3:**
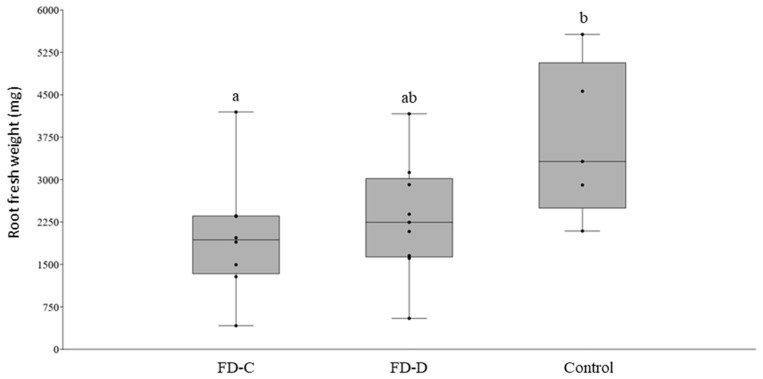
Boxplot with jitter of root fresh weight of Flavescence dorée (FD) infected plants at 120 days post grafting with FD-Cp or -Dp infected scions, in comparison with Control plants grafted with healthy scions. Each dot represents one sample. Different letters indicate significant differences in fresh weigh (ANOVA; F(2, 19) = 3.6627; *p* = 0.045).

To investigate the effects of the two FDp strains on periwinkle root growth, two sets of nine plants were graft-infected with either FD-C or FD-D, together with nine plants grafted with healthy scions. After 120 dpg, root fresh weight of FD-C or -D infected plants was similar, and, in the case of FD-C, significantly lower than that of the control plants (Tukey; *p* < 0.05) (Figure 3).

### 2.3. Effects of the Competition between FD-Cp and FD-Dp Strains in Catharanthus roseus Plants

**Figure 4:**
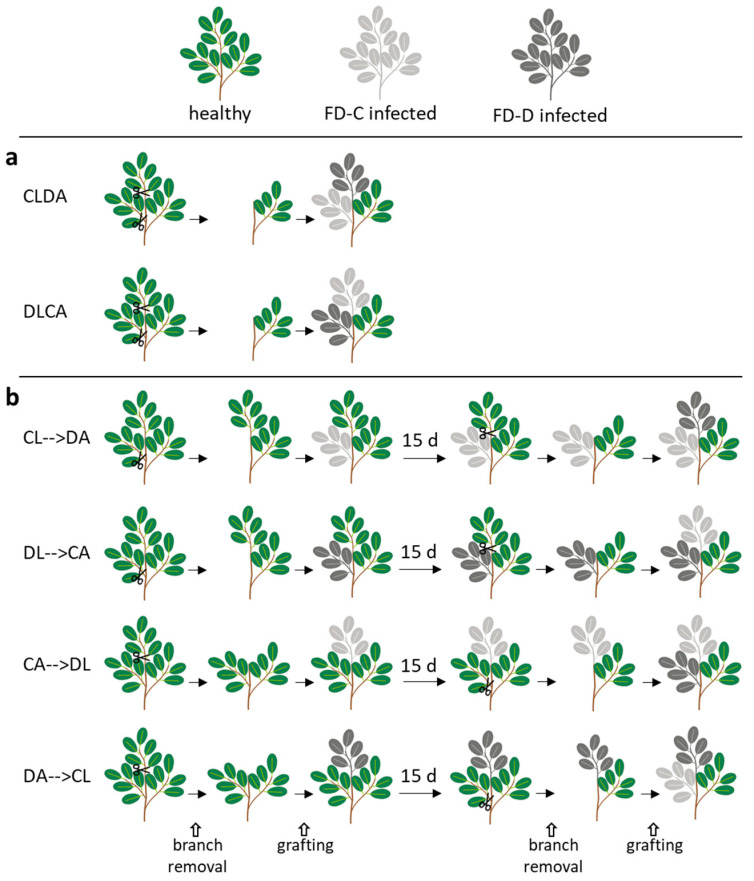
Experimental scheme for grafting scions from healthy (green), Flavescence dorée -C (light gray) and -D (dark gray) strains on periwinkle plants. (**a**) Contemporaneous grafting of either FDp strain (-C or -D) at apical (A) or lateral (L) position (CLDA, DLCA); (**b**) Grafting with temporal advantage: grafting of the first phytoplasma strain (-C, or -D) at apical (A) or lateral (L) position was performed 15 days before grafting of the second strain (-D or -C) at the other position on the same plant (CL→DA; DL→CA; CA→DL; DA→CL).

**Figure 5:**
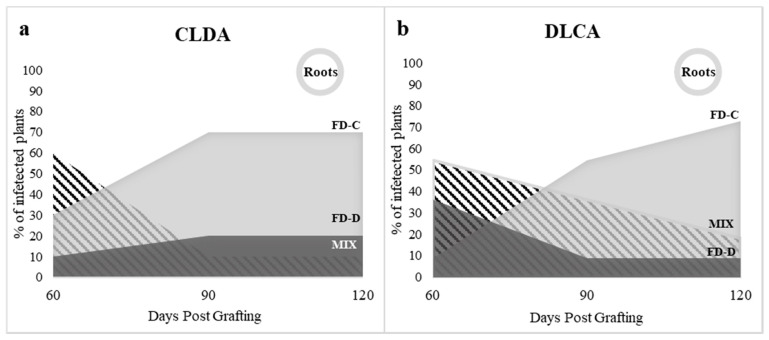
Contemporaneous grafting of either Flavescence dorée phytoplasma (FDp) strain (-C or -D) at apical (A) or lateral (L) position. Percent of single and mixed infections with Flavescence dorée FD-C and -D phytoplasma strains in leaves (graphs) at 60, 90 and 120 days post grafting (dpg) and in roots (circles) at 120 dpg of periwinkle plants with infected scions. (**a**) CLDA: FD-Cp lateral and FD-Dp apical grafting. (**b**) DLCA: FD-Dp lateral and FD-Cp apical grafting. Light gray: FD-C, dark gray: FD-D, black and white striped pattern: mixed infection.

Time course experiments were designed to study the competition between FD-C and FD-D phytoplasmas in colonizing the aerial plant tissues. In the first experimental setting, the two FDp strains were inoculated contemporarily. Two combinations of double grafts were produced to exclude the possibility of an infection advantage due to the apical (A) or lateral (L) graft position (Figure 4a). At 60 dpg, irrespective of the grafting position, six plants were positive for both FDp strains in mixed infection for each CLDA (10 grafted plants) and DLCA (11 grafted plants) experiment (Figure 5, Table S3). The remaining plants were positive for either FD-C or FD-D phytoplasmas in single infections (Figure 5, Table S3). Nevertheless, at 120 dpg, mixed infections decreased (CLDA: 1 plant; DLCA: 2 plants) and the majority of leaf samples (7 and 8 for CLDA and DLCA), as well as all the analyzed roots (n = 16) were positive to FD-Cp in single infection (Figure 5, Table S3). An intermediate situation was found at 90 dpg (Figure 5, Table S3).

**Figure 6:**
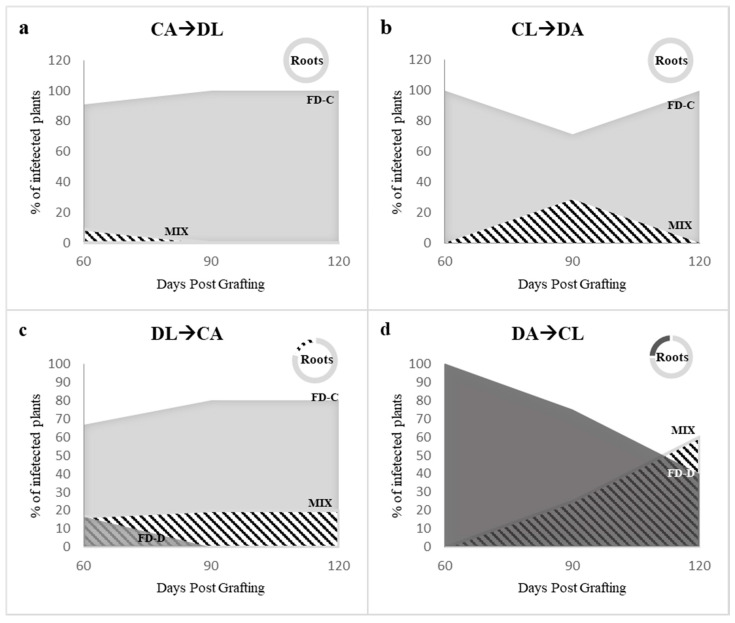
Dual infection with temporal advantage. Percent of single and mixed infections with Flavescence dorée -C and -D phytoplasma (FDp) strains in leaves at 60, 90 and 120 days post grafting (dpg) of periwinkle plants with infected scions (graphs) and in roots (circles) at 120 dpg. (**a**) CA→DL: initial FD-Cp apical grafting followed by FD-Dp lateral grafting 15 days later. (**b**) CL→DA: initial FD-Cp lateral grafting followed by FD-Dp apical grafting. (**c**) DL→CA: initial FD-Dp lateral grafting followed by FD-Cp apical grafting. (**d**) DA→CL: initial FD-Dp apical grafting followed by FD-Cp lateral grafting. Light gray: FD-C, dark gray: FD-D, black and white striped pattern: mixed infection.

In the second experimental setting, a temporal advantage of 15 days was provided to both the FDp strains. Four graft combinations were set up (see Figure 4b). When FD-Cp was the first grafted strain, it was detectable in all plants at 120 dpg, independently from its grafting site (Figure 6a,b, Table S2), while FD-Dp was detected only in mixed infections before 90 dpg in the case of lateral grafting (CA→DL), or up to 90 dpg following apical grafting (CL→DA) (Figure 6a,b, Table S3). FD-Cp strain alone also infected all plant roots at 120 dpg, irrespective of the treatment (Table S4). When FD-Dp was the first grafted strain, the results changed according to the grafting site. When grafted laterally (DL→CA), FD-Dp was detected in single infection only at 60 dpg in one of the 11 grafted plants, while four plants were infected with FD-Cp (Figure 6c, DL→CA; Table S3). At 90 and 120 dpg, FD-Dp was not detected in single infection and present in mixed infection in 2 of the 11 grafted plants, while FD-Cp was detected in single infection in eight plants (Figure 6c, DL→CA; Table S3). At 120 dpg, three of the four plant roots analysed were infected by FD-Cp strain in single infection, and mixed infection was detected in one root sample (Figure 6c, DL→CA; Table S4). When grafted apically (DA→CL), FD-Dp strain was detected at 60 dpg as single infection in the five analysed grafted plants, while at 90 and 120 dpg one and three grafted plants showed mixed infections, respectively (Figure 6d, DA→CL; Table S3). At 120 dpg, FD-Dp strain was detected as single infection in one of the four analyzed roots, while FD-Cp was detected in single infection in the remaining ones (Figure 6d, DA→CL; Table S4).

## 3. Discussion

Multiple infections of pathogen genotypes or species in the same pathosystem can drive disease dynamics and pathogen evolution [23,24]. This finding raised a growing interest in human and animal epidemiological studies; however, less attention has been paid to plant pathosystems. In plants, co-infections are common in both natural and agricultural environments and have the potential to change pathogen accumulation, transmission, and virulence [25].

Different factors, such as plant genetics, environmental variation, and intermediate hosts, can shape the prevalence of co-infection in space and time: combining field surveys and experimental approaches may help to unveil the role of each actor of such a complex scenario. In this work, an experimental design using *Catharanthus roseus* as a model plant was established to shed light on the Flavescence dorée phytoplasma population dynamics observed in infected vineyards of Piedmont [12]. The two phytoplasma genotypes detected in both vines and insects, FD-C and FD-D, were used for controlled inoculation through grafting. Infection dynamics in the different treatments were characterized by means of a new diagnostic protocol developed for differentiation and relative quantification of the two phytoplasmas in mixed infections.

On single infection, FD-C and FD-D strains showed similar virulence, symptomatology, and infection dynamics. Moreover, the time course experiments revealed that the speed and efficiency of colonization of both shoots and roots were comparable. Both phytoplasma strains reached the root system within 12 days from grafting, then spread to the aerial part of the plant, where they could be detected after 45 days together with the first symptom appearance. Indeed, FDp rapidly infects also the root system of the experimental host *Vicia faba* after vector-mediated inoculation of the aerial part by *Euscelidius variegatus* [26]. Rapid movement from the initial inoculation site toward the root system may be a general characteristic of phytoplasma infection of herbaceous hosts, as it has also been reported for ‘*Ca*. P. asteris’ [27]. The two closely related ‘*Ca*. P. asteris’ strains, severe (SAY) and dwarf (DAY), showed instead different infection dynamics; however, the more aggressive behavior of the SAY strain could be a combination of genetic differences in the effector machinery of the two isolates and a possible imbalance in the starting inoculum of the two strains [28]. In our case, a similar amount of both phytoplasmas was inoculated, based on pathogen quantification in the donor plants, and FD-C and -D phytoplasma loads were comparable in the grafted plants up to eight weeks post grafting. Interestingly, SAY titer declined at four weeks from inoculation and, at the end of the observation period, it was below the titer of the mild strain [28]. Similarly, after ten weeks, FD-C phytoplasma titer was significantly lower than that of FD-D. When inoculated together, FD-C generally outcompeted FD-D, even when the latter had a temporal or spatial advantage at grafting. In the few cases in which the two strains coexisted, no differences in symptomatology were observed, suggesting the absence of a synergic effect, which is known in the case of tomato co-infection with Columbia Basin potato purple top and Alaska potato witches’-broom phytoplasmas [13].

The prevalence of FD-C phytoplasma indicates that the interaction between the two strains results in competitive exclusion. Three mechanisms may explain competition between parasites within a host: exploitation, apparent, and interference competition [29,30,31]. For species colonizing the same niche, genetic traits enabling faster or improved use of host resources allow outgrowth of one strain through exploitation competition [31]. This is probably the case of FD-C phytoplasma, which shared similar dynamics of multiplication and host colonization with the FD-D strain under separate inoculation and at early stages of co-inoculation. Improved efficiency of nutrient uptake under limiting conditions can explain the FD-C competitive dominance at late co-infection stages when plant nutrients are depleted, as suggested by Hibbing and co-workers [23] for competing microbes in a resources consumption scenario. The possible occurrence of apparent competition, which is mediated by the host response to the infection [30], may also be considered. If this response is not specific, it may affect other co-infecting pathogens and favor the one that can better escape [31]. Under attack, plants react with oxidative stress as a basal defense mechanism against pathogenic invasions. Proteomic and genetic studies on FDp infected plants [32,33] suggest an increased concentration of reactive oxygen species as consequence of phytoplasma colonization. Consistently, a gene coding for an OsmC-like protein was over-expressed in FD-C infected plants compared to FD-D ones [34]. Osmotically inducible proteins (Osm) are a family of bacterial proteins involved in defense against oxidative stress caused by exposure to organic hydroperoxides [35]. The improved capacity to respond to oxidative stress by FD-Cp could also explain the competitive advantage of this strain over FD-Dp. The experiment in *C. roseus* with temporal advantage seems to rule out a mechanism of competition based on “first come first served” since FD-C strain outgrew FD-D even when grafted afterwards. This phenomenon was particularly evident in the roots. However, interference competition may still be a possible mode of relationship between FD-C and FD-D phytoplasmas. Interference competition among parasites may be based either on the occupation of the available space (priority) or the release of growth-inhibiting compounds (allelopathy) [31]. Obligates intracellular parasites can take advantage of the host cell environment to replicate and spread, and one of their favorite targets is the cytoskeleton, which is exploited for internalization and intracellular movements [36]. In the case of phytoplasmas, infected cells showed a rearranged cytoskeleton [37] and the Immunodominant membrane protein (Imp) of ‘*Ca*. P. mali’ together with the antigenic membrane protein (Amp) of ‘*Ca*. P. asteris’ bind actin filaments of plant and insect cells, respectively [38,39,40]. The Imp gene of FDp is highly expressed [34] and it binds vector proteins [41], among which actin [42]. FD-C and FD-D Imp proteins share less than 60% sequence identity and do not cross-react with specific antisera [43]. Strain-specific competition in actin-binding could also lead to a fitness advantage for FD-C phytoplasma, suggesting that interference competition may act through the occupation of available space (priority, [31]). As for allelopathy, it is known that bacteriocins can target even closely related strains of the same species, leading to interference competition. Although we cannot exclude the production of antimicrobial molecules by FDp, up to now, these genes have not been described in phytoplasma genomes. However, Schneider et al. (2014, [44]) suggest that the type VI secretion system (T6SS), known in gram-negative bacteria to control co-inhabiting parasites [45,46,47], can be also present in gram-positive bacteria and phytoplasmas. In the case of ‘*Ca*. P. mali’, mild strain suppression of aggressive strains is mediated by the AP460 gene, encoding an AAA+ ATPase, a group of proteins that compose the T6SS system [48]. Notably, among AAA+ proteins, eight complete *fts*H genes are described in the FDp genome, with a role in host adaptation [49].

While strain competition in *C. roseus* results in FD-C phytoplasma dominance, in Piedmontese vineyards (Italy) the predominant strain is FD-D [12]. Phytoplasmas are transmitted in a propagative way by phloem-sucking insects [50], and the leafhopper *Scaphoideus titanus* is the vector of FDp. The competitive strategy successful in the plant host may be unsuccessful in the insect one. It is worth noting that the incidence of FD-C strain, even though low in general in the vineyards, is higher in plants than in insects [12]. This evidence suggests that FD-C phytoplasma is favored in the plant host, but the vector may act as a bottleneck, differently shaping the dynamics between FDp strains. Indeed, pathogen competition strategies may differ among hosts, and the relationship between competitive outcome and transmissibility is not straightforward [29]. In any case, different results of the competition in different plant hosts (*V. vinifera* and *C. roseus*) cannot be excluded. The effects of the two FDp strains competing within the vector are currently under analyses, with special regard to colonization of the insect salivary glands. The draft status of FD-C and -D genomes [51,52] does not allow reliable comparison of the two strains, at the moment. Future improvement of the assemblies will show sequence similarities and differences and will help to identify candidate phytoplasma genes for the ‘within-host competition’.

FD is a severe quarantine disease and a threat to vineyard productivity in the most important European wine-producing areas. Control measures are mainly based on large-scale insecticide treatments, with detrimental impacts on public health and benign insects, roguing of infected plants and use of healthy plant material. Therefore, alternative and environmentally sustainable solutions are needed. Unveiling the mechanisms of FDp strain adaptation to different hosts may identify new targets to broaden our control strategies. A better understanding of the molecular bases of the competition between FDp strains may identify mild suppressor strains to outcompete the severe ones. In the case of ‘*Ca*. P. mali’ two mild strains suppress the infection of the aggressive ones both in *C. roseus* and apple trees [44], and are involved in the recovery phenomenon [53]. Recovery is known also in the case of FDp infected grapevines, and, although several approaches have been applied to describe the transcriptomic [54], metabolomic/proteomic [32] and epigenetic status [55] of the recovered plants, the biological and molecular triggers of the process are not understood. Therefore, recovery is still considered to be a “stochastic” event [56], and the influence of the FD genotype is unexplored. For many years, two major FDp genotypes (FD-C and FD-D) were known, but recently further investigations revealed the unexpected genetic complexity of the FDp populations [4,12]. The presence of several minor and previously unknown genotypes is a source of genetic diversity in the perspective of plant protection. Competition of FDp strains for plant colonization encourages further investigations on the exploitation of minor strains for the biological control of the disease.

## 4. Materials and Methods

### 4.1. Phytoplasma Strains and Plant Hosts

The Flavescence dorée 16SrV-C (FD-C; Map-FD3) phytoplasma strain (FD-Piedmont) was isolated in Italy [12,57]. The 16SrV-D (FD-D) phytoplasma strain (FD-D; Map-FD2), was kindly provided by Dr. Xavier Foissac [18]. Both isolates were maintained in periwinkle (*Catharanthus roseus* (L.)) by grafting. Healthy periwinkle plants were obtained by seed and grown in pots in a greenhouse under controlled conditions (22–25 °C; 65–70% RH). For each experiment, periwinkle plants were graft-inoculated and sampled at 30 days post grafting (dpg) to confirm their infection status and to quantify the phytoplasma load. Infected plants with phytoplasma loads between 1.04 × 10^4^ and 4.32 × 10^4^ GU/ng plant DNA were selected as scion donors for the grafting experiments detailed below.

### 4.2. Grafting for Systemic Single or Dual Infection of Catharantus roseus

#### 4.2.1. Dynamics of FD-C and FD-D Single Infections in *Catharanthus roseus*

Time course experiments were designed to study the ability of the two strains to move systemically through the aerial part of the plant and the root system following localized grafting.

Aerial part. Twelve weeks old periwinkles (26 plants) were graft-inoculated with either FD-C or FD-D phytoplasma strains. After 60 days from the grafting, three leaves of each plant were sampled and analyzed by Real Time PCR to determine phytoplasma load.

Roots. Twelve weeks old periwinkles (15 plants) were graft-inoculated with either FD-C or FD-D phytoplasma strains. After 3, 7 and 12 days starting from the grafting, the roots of three plants were sampled and analyzed until FDp was detectable in at least one individual. To investigate the effects of the two FDp strains on periwinkle root growth, nine plants were graft-infected with either FD-C or FD-D phytoplasmas, together with nine plants grafted with healthy scions. At 120 dpg, leaf samples were collected from each grafted plant to confirm the infection status. At the same time, roots were also collected, and their fresh weight was measured.

#### 4.2.2. Dual Infection of FD-C and FD-D Phytoplasma Strains

Two time course experiments were designed to study the competition between FD-C and FD-D phytoplasmas in colonizing the aerial plant tissues. In the first experimental setting, the two FDp strains were grafted contemporarily. Two combinations of double grafts were produced to exclude the possibility of an infection advantage due to the apical (A) or lateral (L) graft position. In particular, two treatments were applied: lateral grafting with FD-Cp and apical with FD-Dp (CLDA, 10 plants), and vice versa (DLCA, 11 plants) (Figure 4a). For top grafting, the apical part of the receiving plant was removed with a horizontal cut using a razor blade. The stem was vertically cut to receive a wedge-shaped scion. For the side grafting, the procedure was similar, but the receiving plant was cut between the second and the third internode with a sharp downward incision. Parafilm was applied to protect the grafted tissues, and plants were covered with a plastic bag for seven days to help maintaining high environmental humidity. The plants were maintained in the greenhouse under controlled conditions (22–25 °C; 65–70% RH). Three leaves per plant were sampled at 60, 90 and 120 dpg for phytoplasma strain-specific detection. At 120 dpg, also roots were sampled for diagnosis. In the second experimental setting, a temporal advantage of 15 days was provided to each of the two FDp strains. At first, each isolate was grafted both apically and or laterally so that four combinations of double grafts were produced (see Figure 4b). In particular, the following treatments were applied: lateral grafting with FD-Cp and, 15 days later, apical with FD-Dp (CL→DA, 10 plants), apical grafting with FD-Cp, then lateral with FD-Dp (CA→DL, 13 plants), lateral grafting with FD-Dp, then apical with FD-Cp (DL→CA, 11 plants), apical grafting with FD-Dp, then lateral with FD-Cp (DA→CL, 10 plants) (Figure 4b). Apical and lateral graftings were performed as described above. The plants were maintained in the greenhouse under controlled conditions (22–25 °C; 65–70% RH). In these cases of non-simultaneous inoculation, the date of the second grafting was considered to be 0 dpg. Three leaves per plant were sampled at 60, 90 and 120 dpg for phytoplasma strain-specific detection. At 120 dpg, also roots were sampled for diagnosis.

### 4.3. Sampling and DNA Extraction

Leaf samples were collected from the top of three branches of each graft-inoculated plant at different sampling times as described above. Total nucleic acids were extracted from 0.5 g of leaves and petioles according to Pellettier et al. [58]. Precipitated and purified DNA samples were re-suspended in 100 μL of Tris-HCl 10 mM pH 8. DNA concentration was measured with NanoDrop 2000 TM Spectrophotometer (Thermo Scientific, Waltham, MA, USA), and all samples were diluted to 20 ng/µL before strain-specific quantitative detection of FDp.

### 4.4. Development of a Strain-Specific Quantitative Assay for Flavescence dorée Phytoplasmas

Three FDp genomic regions were screened as targets for the development of the new diagnostic and quantification protocol based on the discrimination of the melting peak analyses of the amplicons from each FDp isolate: the ribonucleoside-diphosphate reductase 2 β subunit (*nrd*F; partial: MK091399, MK091398 [12]; full length: Xavier Foissac, personal communication [59]), the maltose ABC transporter membrane subunit (*mal*G; partial: MH547713, MH547714 [12]; full length: Xavier Foissac, personal communication [59]), and the group II intron gene (contig 12) [34]; Xavier Foissac, personal communication [59]). Primer pairs for each target locus were designed on corresponding blocks of conserved sequences, and used in Real Time PCR reactions (Table 1). Each reaction mixture (10 µL final volume) was performed with iTaq Universal SYBR Green Supermix (Bio-Rad) on a CFX-BioRad, in the presence of 300 nM of each primer, and 40 ng of total nucleic acid extracts from FD-C or FD-D-infected periwinkles. Each sample was run in triplicate on the same plate. Cycling conditions were as follow: initial denaturation at 95 °C for 3 min, followed by 45 cycles of 10 s at 95 °C and 30 s at 59 °C. At the end of the PCR, a final melting curve analysis with an incremental temperature of 0.1 °C per cycle was performed, to distinguish amplicons from the two FDp strains based on the differential temperature of their melting peaks.

For the development of a quantitative strain-specific protocol based on TaqMan chemistry, a duplex Real Time PCR was designed on *nrd*F genes. The primers NrdF_F28 and NrdF_R121 were designed between positions 28 and 121 after the gene starting codon and they matched both FD-C and -D sequences. On the amplified sequence, two specific TaqMan probes for *nrd*F -C or -D were designed on a portion displaying two SNPs between FD-C and FD-D phytoplasma strains (Table 1). Specificity of each probe was confirmed using DNA extracts from *C. roseus* infected with either one of the two FDp strains, and on DNA extracted from healthy plants, as negative control. Two microliters of each diluted sample were used as template for qPCR that was performed with iTaq Universal Probes Supermix (Bio-Rad) on a CFX-BioRad. Each sample was run in triplicate on the same plate. Cycling conditions were as follow: initial denaturation at 95 °C for 3 min, followed by 45 cycles of 10 s at 95 °C and 30 s at 60 °C. To obtain two standard plasmids for FDp quantification, *nrd*F-C and -D genes were amplified from total DNA extracted from infected periwinkles with the primers NrdF_F20 (5′-TGCATAAATTTCACGGAGCT-3′)/NrdF_R (5′-TAACGGACAAAAGCGTTTAC-3′) using standard PCR conditions. After purification of PCR products with the DNA Clean & Concentrator Kit (Zymo Research), the amplicons were ligated into the pGEM-T easy cloning vector following the manufacturer’s instructions (pGEM-T- clone kit, Promega, Madison, WI, USA). After transformation of *E. coli* DH5α competent cells by heat shock, recombinant plasmids were extracted using the Wizard SV Plus Minipreps DNA Purification System (Promega, Madison, WI, USA) and quantified with NanoDrop 2000 TM Spectrophotometer (Thermo Scientific, Waltham, MA, USA). For absolute quantification of the two FDp strains, two standard curves were obtained from recombinant pGEM plasmids harboring a fragment of the corresponding targets (pGEM-NrdF-C; pGEM-NrdF-D), serially diluted from from 100 pg to 10 fg, corresponding to 10^7^ to 10 genomic units (GU) of FDp. Standard curves were constructed with the CFX Manager™ Software, by linear regression analysis of the Cq value of each standard dilution replicate over the log of the number of plasmid copies present in each sample. Moreover, to verify the reliability of the probes in discriminating between the two strains when present simultaneously in the DNA sample, the two standard plasmids were mixed at different concentration ratio (1:1; 1:5; 1:7.5; 1:10) that were used as template for the qPCR.

### 4.5. Statistical Analyses

PAST software was adopted for statistical analysis [60]. Analysis of variance (ANOVA), followed by Tukey post hoc test, was used for shoot phytoplasma load and fresh root weight comparisons. T-test was adopted for the comparison of root phytoplasma loads.

## Figures and Tables

**Table 1:** List of primers and probes developed in this study for the simultaneous detection of 16SrV-C and -D Flavescence dorée phytoplasma strains. Target genetic loci, Real Time chemistry of the assay, primer and probe names, sequences, amplicon sizes, number of SNPs in the amplicons, annealing temperature of the assay (TA), Real Time PCR parameters (melting temperature of the amplicons, MT, for SYBR Green assays or R^2^ and reaction efficiency for the TaqMan assays) are provided. MT_-C_, MT_-D_: melting temperature of 16SrV-C or -D amplicons, respectively.

Locus	Chemistry	Name	Sequence (5′-3′)	Size (bp)	N° of SNP	TA (°C)	qPCR (MT_-C_; MT_-D_)	qPCR (R^2^; Efficiency)
*nrd*F	SYBR Green	nrdF_Real_F	AGATGACTTATTTCAATGGGGCG	173	4	59	72.10 °C ± 0.14;	-
		nrdF_Real_R	CAGAAGCGACCATTGCTTTCCA				72.25 °C ± 0.07	
	Taq Man	nrdF_F28	TTTCACGGAGCTAATTGGAA	94	2	60	-	-
		nrdF_R121	TGATATCTTCTGGACGCCA					
		nrdF -C	HEX-TGAAGATGAATATACCCAATATTTTTACGAACA-BHQ1	-	-	-	-	0.996; 99%
		nrdF -D	FAM-TGAAGATGAATATACTCAATATTTTTATGAACA-BHQ1	-	-	-	-	0.999; 101%
*mal*G	SYBR Green	malG_F	GCTTTCCGAGGCCAATTCCA	267	6	59	75.70 °C ± 0.00;	-
		malG_R	ATTCTGGCCAAGCATAAGCG				76.05 °C ± 0.07	
contig 12	SYBR Green	contig12Fw3	AGTTTGATCCAGCTTGCGGA	141	5	59	77.74 °C ± 0.05;	-
		contig12Rv2	CGGCAACTGTGTAAATCCGT				78.08 °C ± 0.04	

## Supplementary Materials

The following are available online at https://www.mdpi.com/2223-7747/9/11/1594/s1, Table S1: Time course of root infections; Table S2: Root weights; Table S3: Numbers of infected leaf samples; Table S4: Numbers of infected root samples.
