# Supplementary material for: Competitive Exclusion of Flavescence dorée Phytoplasma Strains in Catharanthus roseus Plants
Source: Plants (Basel). 2020 Nov 17;9(11):1594. doi: 10.3390/plants9111594 (PMC7698599; doi:10.3390/plants9111594)
Supplement: Supplementary file 1 [file plants-09-01594-s001.pdf]

FDp strain	dpg	Roots tested (n)	FD-positive roots (n)	Phytoplasma titer (FDp GU/ng plant DNA)	Phytoplasma min/max titer (FDp GU/ng plant DNA)
FD-C	3	3	0	-	-
	7	3	0	-	-
	12	9	6	8.92E+04 ± 4.04E+04	2.32E+03/2.31E+05
FD-D	3	3	0	-	-
	7	3	0	-	-
	12	9	8	4.40E+05 ± 2.20E+05	3.75E+03/1.69E+06

Table S1 **Time course of root infections.** Infection rate of *Catharanthus roseus* roots infected by FD-C or FD-D strains, phytoplasma titre (mean±SEM) and phytoplasma titre range observed in the time course experiment to study the root colonization dynamic of the two phytoplasma strains. dpg= days post grafting.

FDp strain	Roots weights (grams)	Root weights range (grams)
FD-C	2.00±0.39	0.42/4.2
FD-D	2.31±0.39	0.55/4.16
Control	3.69±0.62	2.09/5.57

Table S2 **Root weights**. Weights (mean±SEM), weight range (minimum and maximum), phytoplasma titer (mean±SEM), and phytoplasma titre range of *Catharanthus roseus* roots infected by FD-C or FD-D 120 days post grafting.

LEAVES 60 dpg						
	FD-C	FD-D	MIX	Negative	Dead	tot
CLDA	3	1	6	0	0	10
DLCA	1	4	6	0	0	11
CL→DA	6	0	0	2	2	10
DL→CA	4	1	1	4	1	11
CA→DL	10	0	1	0	2	13
DA→CL	0	5	0	0	5	10
Total	24	11	14	6	10	65
%	37%	17%	22%	9%	15%	

LEAVES 90 dpg						
	FD-C	FD-D	MIX	Negative	Dead	tot
CLDA	7	2	1	0	0	10
DLCA	6	1	4	0	0	11
CL→DA	5	0	2	0	3	10
DL→CA	8	0	2	0	1	11
CA→DL	10	0	0	0	3	13
DA→CL	0	3	1	1	5	10
Total	36	6	10	1	12	65
%	55%	9%	15%	2%	18%	

LEAVES 120 dpg						
	FD-C	FD-D	MIX	Negative	Dead	tot
CLDA	7	2	1	0	0	10
DLCA	8	1	2	0	0	11
CL→DA	7	0	0	0	3	10
DL→CA	8	0	2	0	1	11
CA→DL	7	0	0	0	6	13
DA→CL	0	2	3	0	5	10
Total	37	5	8	0	15	65
%	57%	8%	12%	0%	23%	

Table S3 **Numbers of infected leaf samples** at 60, 90 and 120 days post grafting (dpg), and Flavescence dorée phytoplasma strain, according to the experimental conditions.

	FD-C	FD-D	MIX	Total
CLDA	8	0	0	8
DLCA	8	0	0	8
CL→DA	3	0	0	3
DL→CA	4	0	1	5
CA→DL	6	0	0	6
DA→CL	3	1	0	4
Total	32	1	1	34
%	94%	3%	3%	

Table S4. **Numbers of infected root samples** at 120 days post grafting, and Flavescence dorée phytoplasma strain, according to the experimental conditions.
